# An Automatic Assessment System for Alzheimer’s Disease Based on Speech Using Feature Sequence Generator and Recurrent Neural Network

**DOI:** 10.1038/s41598-019-56020-x

**Published:** 2019-12-20

**Authors:** Yi-Wei Chien, Sheng-Yi Hong, Wen-Ting Cheah, Li-Hung Yao, Yu-Ling Chang, Li-Chen Fu

**Affiliations:** 1 National Taiwan University, Department of Computer Science and Information Engineering, Taipei, Taiwan; 2 National Taiwan University, Department of Psychology, Taipei, Taiwan

**Keywords:** Population screening, Quality of life

## Abstract

Alzheimer disease and other dementias have become the 7th cause of death worldwide. Still lacking a cure, an early detection of the disease in order to provide the best intervention is crucial. To develop an assessment system for the general public, speech analysis is the optimal solution since it reflects the speaker’s cognitive skills abundantly and data collection is relatively inexpensive compared with brain imaging, blood testing, *etc*. While most of the existing literature extracted statistics-based features and relied on a feature selection process, we have proposed a novel Feature Sequence representation and utilized a data-driven approach, namely, the recurrent neural network to perform classification in this study. The system is also shown to be fully-automated, which implies the system can be deployed widely to all places easily. To validate our study, a series of experiments have been conducted with 120 speech samples, and the score in terms of the area under the receiver operating characteristic curve is as high as 0.838.

## Introduction

According to the latest statistics published by the World Health Organization, Alzheimer disease and other dementias have become the 7th cause of death worldwide and is costing the society about $818 billion, which amounts to 1.1% of the global gross domestic product (GDP)^1^. There is around 50 million people around the world suffering from dementia, and more than 90% among them are aging more than 65 years old^2^. While there are many types of dementia, and each differs from the others in terms of its pathology and symptoms, Alzheimer’s Disease(AD) would be our focus in this study because it is the most prominent among all.

AD is characterized by progressive declines in multiple cognitive function^3^, which includes memory impairment and at least one of the following: aphasia, apraxia, agnosia or disturbance in executive functioning. Social or occupational function is also impaired because of these deficits. Some signs and symptoms can be found in early stage of AD, which includes memory loss that deteriorates life quality, difficulties in planning and dealing with familiar tasks, confusion with the present state, trouble in visual perception, poor word finding ability and usage in speaking or writing, bad decision-making, and change in mood and personality. Most of these signs can be observed through verbal activities of the patients since language is one of the most advanced forms of the cognitive function we possess and the fundamentals of expressing thoughts. Studies have also shown that features of the preclinical AD include poor word-finding ability, abstract reasoning, and memory^4,5^, which can also be inferred verbally.

As world ages, the growth rate of the AD is exponential and showing no sign of stopping. It is expected that 5.7 million Americans are living with AD, and 5.5 million for people age 65 and older. The situation is even more critical in Taiwan, which is projected to become a “super-aged society” in the next seven years^6^, and thus becoming a high-risk country for AD. As a disease without a cure, there is only a handle of treatments to slow down the deterioration. To maximize the effectiveness of the treatment and to extend the time of quality life of the patients, studies have shown that early intervention is critical for the benefits of the patients and their caregivers^7,8^. However, the increasing number of patients has become a huge burden for the healthcare system, which is already having problems with timely diagnosis. Thus, a system that is able to assess the dementia status of a person is indispensable.

To assess the cognitive status of a person, his/her speech is clearly the most easily accessible data to be analyzed compared with other medical approaches, which are somewhat uncomfortable for the patients and yield no more decisive test outcomes^9–11^. The current literature on systems which detect or assess the status of people with AD based on speech signal analysis can be divided into two main categories considering the feature set used. One is a class of systems that work with context-dependent features^12–14^ and another is with acoustic-dependent features^15–18^. Context-dependent-features are those that are related to the meaning, grammar, or logic of the speech. An ASR is almost always required for the generation of the spoken content before analysis on the relevant context could be carried out. Systems utilizing these features could often achieve the highest performance because they are extremely information-rich, but the robustness is highly depending on the quality of the ASR. On the other hand, acoustic-dependent-features, on the other hand, are those that do not require the understandings of the contents. These include rhythm, intonation, frequency, *etc*. Most of the derived features are based on statistics or self-defined algorithms. The advantages of a system incorporating these features are little effort is needed to apply the methodology to another language. However, the overall analytical process is often complex as a separate algorithm is necessary to deal with each type of explored feature.

Besides, one of the common procedures among the existing literature no matter it is a context-dependent-feature-based system or an acoustic-dependent-feature-based system was the feature selection/filtering process. An excessive number of statistical features would first be derived, and those with higher significance would then be selected and used to train a classifier, which is deemed as a highly inefficient process. Also, the type of classifier used in the existing literature were mainly the support vector machine (SVM), random forest, regression models. Artificial neural network models are not heavily relied on in spite of its recent success in a variety of application, which leaves the question whether it could potentially outperform the current state-of-the-art method.

Therefore, in this study, a novel Feature Sequence representation for characterizing speech data from patients with Alzheimer’s disease in a neuropsychological test scenario is proposed. Three types of neuropsychological test were selected in our system based on the neuropsychological assessment carried out in a clinical setup. An Alzheimer Disease Assessment Engine based on a bidirectional recurrent neural network with gated recurrent unit is trained to carry out the classification. Moreover, we have shown that the Feature Sequence can be generated automatically with the help of the Feature Sequence Generator, which is based on a deep convolutional recurrent neural network trained with the connectionist temporal classification loss. Cross validating with a total of 120 samples, which half of them were from the cognitive healthy subjects and the others were from the Alzheimer’s disease subjects, an area under the receiver operating characteristic curve score of 0.838 is achieved.

## Methods

### System overview

**Figure 1:**
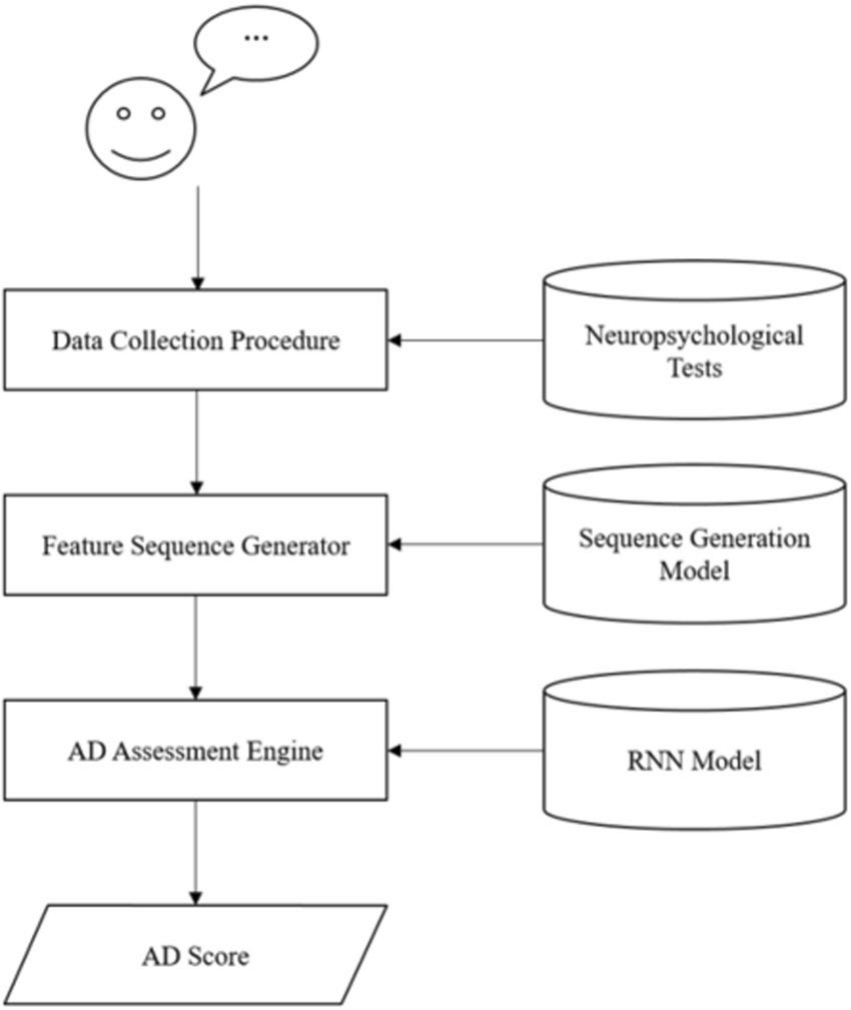
The flowchart of the system.

There were mainly three components in our system: a data collection procedure that collected the speech data from the user, the Feature Sequence Generator that turned the speech data into the Feature Sequence, which was a novel feature representation proposed in this study, and an AD Assessment Engine that generated the score of having AD. The flowchart of the system is shown in Fig. 1.

### Data collection procedure

Data collection procedure of our system was as follow. First, the user was being instructed about the form and the flow of the subsequent neuropsychological tests in person. Next, the user was asked to sit at a desk placed with a microphone and a pair of speakers on top. With a total of six selected neuropsychological tests per session, the description of each neuropsychological test was played for 30 seconds, and was followed by an answering window of 1 minute. Speech data were only recorded during that one-minute period. Overall, it only took less than 10 minutes to complete a session.

### Neuropsychological test selection

Three types of neuropsychological test were selected in our system, which were the fluency test^19–21^, the picture description test^22–24^, and the logical memory test^25–27^. The selection was based on the neuropsychological assessment carried out in a clinical setup as well as well-known research regarding AD. These tests had been proven to be effective in characterizing the key features of AD, which were all very differentiable problems.

#### Fluency test

The proceeding of the fluency test was as follows. Given a designated category, the subject was asked to say as many different words related to that category as possible for a limited time. For example, if the category was animal, possible answers were elephants, tigers, zebras, *etc*. In our system, the category of animal and fruit were picked, and the time limit for each category was one minute.

#### Picture description test

The proceeding of the picture description test was as follows. Given a presented picture, the subject was asked to describe the scenario in the picture as detailed as possible for a limited time. For example, the following description was a possible answer. This is a picture showing a typical evening of a family. Daddy is watching baseball, and mommy is knitting a scarf. The current time is …, *etc*. In our system, a picture from a related study conducted in Japan^28^, which shared a similar cultural background with us, and another from the Western Aphasia Battery (WAB)^29^ are picked. The time limit for each picture was one minute as well.

#### Logical memory test

The procedure of the logical memory test was as follows. A short story was read out loud to the participants, and after that the subject was asked to spontaneously recall the story as precise as possible. In our system, two stories of the Wechsler Memory Scale III (WMS-III)^30^ were included for analysis. Although there was no time limit for logical memory tests in a clinical setup, a time limit of one minute was still applied in our system for the sake of consistency.

### Feature representation – feature sequence

Unlike many of the existing literature^12–18^, our goal was to design a representation that could implicitly embody features all at once. What we came up with was a sequence of tokens, where each token was responsible for representing one unique element in the speech. Using such a representation, pausing could be identified by a silence token or a filled pause token, repeating could be identified by tokens that recur throughout the sequence, and disfluency can be identified by the alternating occurrence between silence tokens and other tokens. Besides these three key characteristics, many other useful derived features discussed in existing literature^13^ can also be identified. For example, length and speed of the speech were both proportional to the number of tokens in the sequence. Even some of the context-dependent features such as number of unique word and low-frequency word could have the potential of being inferred from the distribution of tokens because each token may be a pretty close approximation to a specific word. These properties made our design fully capable of portraying the speech of AD patients, which was an excellent candidate for tasks like detection and assessment of AD. The sequence of tokens is referred to as the Feature Sequence in the following contents.

To meet the criteria of the Feature Sequence, the possible candidate tokens were phonemes, syllables, words, utterances, *etc*. The primary consideration of selecting the optimal candidate was suitability, trainability, and generalizability. Suitability was how appropriately the tokens could manifest the key characteristics. This ruled out phoneme since it is the minimum units of speech, so there were going to be a lot of recurrent tokens, which might be misleading in identifying repeating as that might originate from either the same word or different words. Trainability was how feasible it was to build a classifier for AD assessment based on the Feature Sequence and an automatic Feature Sequence generator. This ruled out utterance since it was impossible to collect enough training data. Finally, generalizability was how well the tokens could incorporate regional dialects and the mixed-language usage in Taiwan. This ruled out word since there is not even an official written form for Taiwanese and Hakka. On the other hand, the syllables of Mandarin Chinese, Taiwanese, and Hakka are very similar, which made it generalizable.

Therefore, syllables were the choice of our tokens. Furthermore, as Mandarin Chinese, Taiwanese, and Hakka being a monosyllabic language, syllable alone can contain much information about the spoken content. This is a valuable upgrade to the Feature Sequence because it was competent to embody both acoustic-dependent and context-dependent features. ZhuYin, the most popular way of spelling in Taiwan, was used to define the token space for our Feature Sequence. According to the dictionary maintained by the Ministry of Education of Taiwan, there are 1339 different syllables spelled in ZhuYin, and only 317 among those are retained after discarding tone markers, i.e., ´, ˇ, `, ˙, and similar phonetic units, i.e., , to increase trainability by decreasing the token space; additionally, generalizability would also be increased because less adverse effect caused by different accents would arise because different tones were now grouped together and viewed as one single unit. A silent token is also added to the token space. However, because there is only one token indicating silence in the token space, a threshold for judging whether a silence segment should be transcribed to a silence token based on its length needed to be determined. Eventually, the token space for the Feature Sequence was 318.

### Feature sequence generator

Given the collected speech data as the input, there were two ways of generating the Feature Sequence: one was done manually by human labeling whereas the other was done automatically by a model. The model of choice was a Convolutional Recurrent Neural Network (CRNN) trained by Connectionist Temporal Classification (CTC) loss^31^.

#### Model architecture

**Figure 2:**
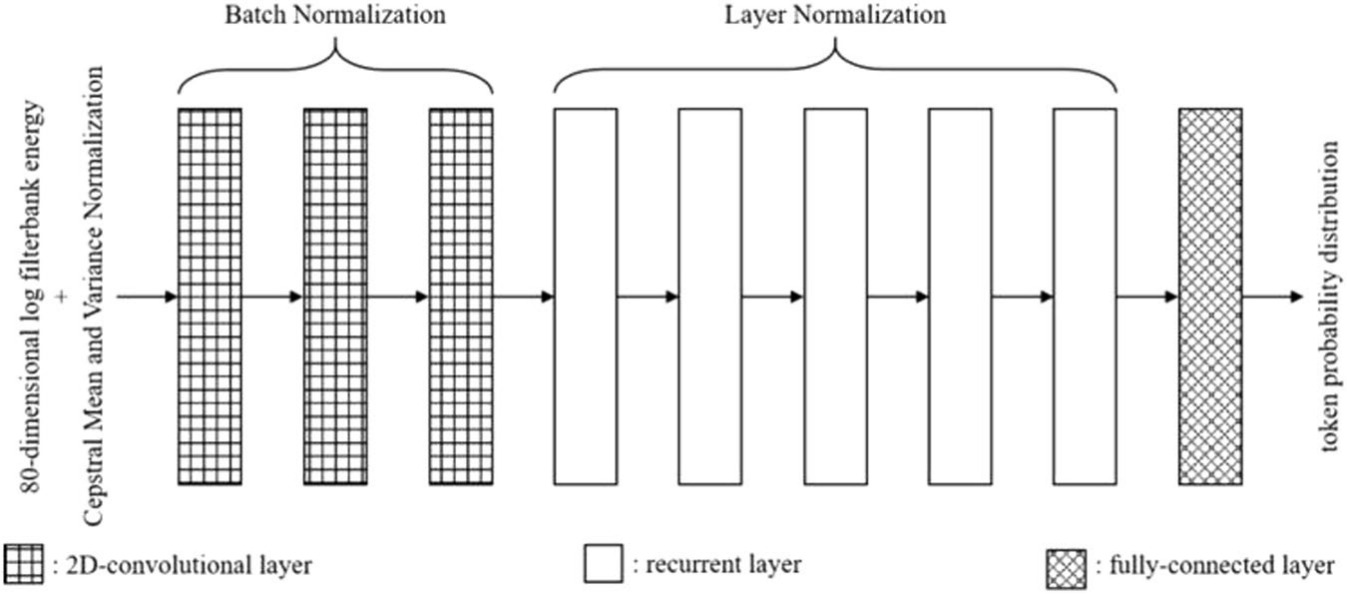
The block diagram of the Feature Sequence Generator.

In our system, the input of model was the 80-dimensional log filterbank energy extracted with a window size of 25 ms and a hop size of 10 ms. Moreover, Cepstral Mean and Variance Normalization (CMVN)^32^ was applied to normalize the derived energies. The architecture of the Feature Sequence Generator was mostly inspired by the state-of-the-art end-to-end speech recognition model evaluated on both English and Mandarin Chinese, namely the Deep Speech 2 from Baidu, Inc.^33^. Some key highlights of the Deep Speech 2 are summarized as follows. First, a bidirectional Recurrent Neural Network(RNN) setup would hugely improve the performance of the model. Second, applying 2D convolution layers prior to the RNN could reorganize the spectrogram by modeling the temporal translation invariance and the spectral invariance and reduce the computation cost of CTC loss by scaling down the number of timesteps. Finally, applying Batch Normalization (BN)^34^ to every layer increases training speed and further boosts the performance of the model. However, with limited hardware resources at hand, the Feature Sequence Generator had 3 2D-Convolutional layers, followed by a 5-layered bidirectional RNN, and finally a fully-connected layer. For the Convolution layers, the number of the filters was 32, 32, and 96 respectively, the kernel size of the filters was (11, 41), (11, 21), and (11, 21) respectively. For each layer of the RNN, there were 512 GRU cells in both directions. For the fully-connected layer, there were 318 (correspond to the number of classes in the token space of the Feature Sequence) + 1 (correspond to the “blank” token) nodes, and the activation function is a softmax function. BN is also applied in all 3 convolution layers right before the activation. However, rather than applying BN implemented in Deep Speech 2, Layer Normalization (LN) is applied in all 5 RNN layers because LN seems to be more suitable than BN when dealing with RNN applications^35^. The block diagram of the Feature Sequence Generator is shown in Fig. 2.

#### Model training

To train the Feature Sequence Generator, four datasets in Mandarin Chinese were collected, which were Aishell^36^, Primewords Chinese Corpus Set 1^37^, Free ST Chinese Mandarin Corpus^38^, and THCHS-30^39^. It added up to a total of 307961 instances and 422 hours of data. The average duration of each instance was 7 seconds, and all instances over 10 seconds were removed from the training set because longer input sequences have a greater chance in facing the issue of vanishing and exploding gradient. Backpropagation through time (BPTT) was carried out using Adam^40^ with a learning rate of 0.0005 as the optimizer. Gradient clipping was also applied to further stabilize the training, where the maximum allowable gradient was 400. The batch size was set to 32 throughout the whole training process, and the model was trained for 50000 iterations. All weights were initialized using Glorot normal initializer^41^.

#### Feature sequence generation strategy

Generating the Feature Sequence was generally done by greedy decoding^42^. All tokens excepted the silence token could be generated by selecting the one which had the maximum model output at each timestep. In order to generate the silence token, a simple heuristic was designed to determine the length of the silence segment and whether to generate a silence token based on the determined length. First, the blank token output by the Feature Sequence Generator was treated as a silence or at least nothing significant. Then, only a number above a certain threshold of consecutive blank tokens would be transcribed to a silence token. The threshold could be specified in terms of seconds, i.e., how many seconds should one silence segment be in order to be treated as a silence token. Because the hop size of the input was 10 ms, a silence token would only be transcribed when there exists at least the threshold (in terms of seconds) divided by 0.01 of consecutive blank tokens. For example, given the threshold is 3 seconds, a silence token would only be transcribed when there exist at least 300 consecutive blank tokens.

### Alzheimer’s disease assessment engine

Given the Feature Sequence as the input, which implicitly contains the necessary information for assessment, the output was the assessment score of having AD. We formulated the score of having AD with a function of a set of tokens in the Feature Sequence, as shown in the following equation:

1 $$score=f({s}_{1},\,{s}_{2},\,\ldots ,\,{s}_{T})\in [0,\,1]$$

where *s*_*t*_ is the *t*^*th*^ token in the Feature Sequence, and *T* is the maximum length of the Feature Sequence. The assessment score is a scalar value ranging from 0 to 1, where the higher the assessment score is, the higher the chance of having AD. Instead of handcrafting abundant features and selecting the significant ones via statistical analysis afterwards to train a classifier, data-driven machine learning technique is utilized to build our classifier. The model of choice is a RNN.

#### Model architecture

At a higher-level standpoint, RNN can also be generally formulated as:

2 $${h}_{t+1},\,{y}_{t}=RNN({x}_{t},\,{h}_{t})$$

where *x*_*t*_ is the input of timestep *t*, *y*_*t*_ is the output of timestep *t*, and *h*_*t*_ is the RNN’s hidden state of timestep *t*. It is a perfect fit for our problem since its strength is sequence modeling. The similarity can also be seen by comparing Eqs. (1) and (2) We believe after a RNN has processed the Feature Sequence by substituting *x*_*t*_ into *s*_*t*_, the output from its final timestep, which can also be viewed as an encoded message or a feature vector of the whole sequence, would have enough information for classifying through a fully-connected layer, that is,

3 $$score=\sigma (W{y}_{T}+b)$$

where *y*_*T*_ is the RNN output of the final timestep, *W* is the weight, *b* is the bias, *σ* is the activation function of the fully-connected layer, and score is the assessment score of having AD.

**Figure 3:**
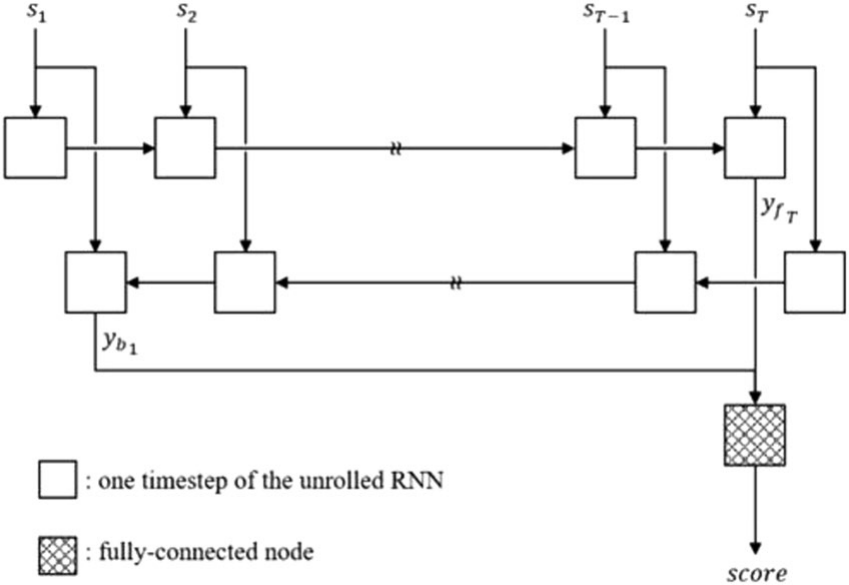
The block diagram of the AD Assessment Engine.

With only limited data at hand, the architecture of the AD Assessment Engine is designed to be as lightweight as possible to increase trainability and decrease the chance of overfitting by limiting its capacity. Eventually, it is a single-layered bidirectional RNN with 128 GRU cells in each direction, and the output from the final timestep in each direction is concatenated and fed through a fully-connected layer to generate the final output, where it is a single scalar value ranging from 0 to 1. The activation function of the GRU output is a tanh, and that of the GRU gate control and the fully-connected output is a sigmoid function. The block diagram of the AD Assessment Engine is shown in Fig. 3.

#### Model Training

Since the output of the AD Assessment Engine was activated by a sigmoid function, it ranges from 0 to 1 and could be treated as a probability. The corresponding label for each output was thus 0 for subjects without AD and 1 for subjects with AD. The loss function was defined as the cross entropy sum between the output and the label of all training samples in a batch. BPTT is carried out using Adam with a learning rate of 0.001 as the optimizer. The batch size is set to 16 throughout the whole training process. All weights are initialized by using the Glorot normal initializer^41^.

### Data preparation

The presented study was approved by the Ethics Committee and Institutional Review Board at the National Taiwan University Hospital. The collection of data and all methods in this study were all performed in accordance with the approved guidelines and regulations. Written informed consent was obtained from all participants.

#### Mandarin_Lu & NTU dataset

Mandarin_Lu corpus from the DementiaBank is a dataset containing interview recordings from 52 AD patients in Taiwan^43,44^. In order to match the data collected using our data collection procedure, the data were augmented manually by segmenting the first-minute response from the subject. Only 30 subjects from the dataset were selected because the rest was either shorter than one minute or considerably interfered by the interviewer. The selected data include three neuropsychological tests, which are a fruit fluency test, a location fluency test, and a picture description test using the picture from WAB. Using the data collection procedure stated above, another 30 cognitive healthy (CH) subjects were recruited on our own as a control group under an institutional review board approval from the National Taiwan University Hospital. The neuropsychological tests used during our collection is exactly the same as the ones selected from the Mandarin_Lu corpus. This dataset is named NTU dataset. The number of samples in the Mandarin_Lu and NTU dataset for both the fruit and location fluency test are 30 and that for the picture description test are 15.

#### NTUH Dataset

While a combination of Mandarin_Lu dataset and NTU dataset was used to pilot-study the proposed system, the overall difficulty of the task is not as hard because the two test groups are quite different from each other in terms of their cognitive abilities. Moreover, the recording environment and the quality of the recording are much different as well. Finally, there is no access to the subject’s medical report as well, so it is uncertain whether some other complications occurred alongside AD. To overcome the weakness of Mandarin_Lu dataset and NTU dataset, twenty subjects were further recruited using the data collecting procedure stated above, where 10 subjects are CH and 10 subjects are AD. This dataset is named NTUH dataset. The diagnosis of mild AD was based on the NINCDS-ADRDA Alzheimer’s criteria. Participants were excluded if they had current or past diagnosis of a psychiatric disorder, alcohol or drug abuse, learning disability, known head injury with loss of consciousness, untreated hypothyroidism, Vitamin B12 deficiency, metabolic derangement, or any significant visual or auditory impairment that precluded participation in neuropsychological testing. With 6 neuropsychological tests per subject, there were 120 one-minute samples in total. Table 1 lists the demographics of the subjects in NTUH Dataset. Subjects were recruited on our own as a control group under an institutional review board approval from the National Taiwan University Hospital.

**Table 1:** Subject information of NTUH Dataset.

	CH	AD
Number of data	10	10
Age	67.2 ± 8.42	78.6 ± 6.49
Years of education	16.2 ± 1.88	13.0 ± 3.46
MMSE	28.6 ± 0.91	22.4 ± 3.72
Gender(% women)	60	40

## Results

### Evaluation of the Alzheimer’s Disease Assessment Engine

To examine the efficiency of modeling the speech pattern from AD patients using the Feature Sequence and RNN, a series of experiment on different RNN cells and recurrent directions were conducted. The Feature Sequence used in the current section was based on the manual transcription of Mandarin_Lu corpus generated by human labeling rather than the automatic transcription generated by the Feature Sequence Generator. To avoid overfitting and prove the effectiveness of our end-to-end design, all experiments were carefully conducted using the following evaluation procedure. First, data were randomly shuffled so that samples from the two classes were fully mixed. Next, 10 samples were selected at a time sequentially to be the testing set through the mixed data. The unselected data were split into a training set and validating set with the ratio of 85:15. Note that the data were shuffled at the beginning, so the expected number of samples from the two classes was equal in all three sets. Model training was carried out using only the training set, and it was stopped when the validation loss failed to improve for 20 consecutive epochs. The best model was selected according to the lowest validation loss throughout the training process and was then used to evaluate the outcome of the testing set. Such a procedure was repeated for a number of times until every sample in the dataset had been tested. The outcome from each testing set were aggregated, and a score of the area under the receiver operating characteristic curve (AUROC) is obtained. A completion of such a procedure was called a trial. To further minimize the influence due to the randomness of training Artificial neural network(ANN), six trials were carried out and the AUROC scores were averaged to get the overall AUROC score.

#### Comparison between different RNN cells

Three types of commonly used RNN cell units were put to test, which were the GRU cell, the LSTM cell, and the simple cell. The number of cells used were all 256, and the RNNs were unidirectional. The Performance are shown in Tables 2–4, where the performance of the simple cell was the worse, and that between the GRU and the LSTM cell was matched with the former having a slight edge over the latter. It showed that the gating mechanism as well as the additional cell states in the GRU cell and the LSTM cell truly helped a lot in modeling longer sequence. This would be a crucial factor when dealing with longer Feature Sequence presented in neuropsychological tests such as the picture description test. For the consistency with the Feature Sequence Generator and the slight performance edge, the GRU cell was preferred in building the AD Assessment Engine to encode the Feature Sequence. Moreover, there are less parameters to be trained in the GRU cell compared with that in the LSTM cell, which would increase both the training speed and the training stability.

**Table 2:** The AUROC score on different RNN cell unit.

	Unidirectional			Bidirectional		
	GRU	LSTM	Simple	biGRU	biLSTM	biSimple
thr = 1	0.932 ± 0.02	0.922 ± 0.01	0.682 ± 0.08	0.951 ± 0.02	0.948 ± 0.02	0.871 ± 0.06
thr = 3	0.941 ± 0.03	0.916 ± 0.02	0.758 ± 0.06	0.969 ± 0.01	0.942 ± 0.01	0.888 ± 0.03
thr = 5	0.936 ± 0.02	0.922 ± 0.03	0.760 ± 0.03	0.948 ± 0.01	0.937 ± 0.02	0.885 ± 0.02

**Table 3:** The Sensitivity score on different RNN cell unit.

	Unidirectional			Bidirectional		
	GRU	LSTM	Simple	biGRU	biLSTM	biSimple
thr = 1	0.862 ± 0.04	0.869 ± 0.02	0.624 ± 0.05	0.891 ± 0.03	0.853 ± 0.03	0.771 ± 0.06
thr = 3	0.889 ± 0.01	0.889 ± 0.03	0.702 ± 0.07	0.887 ± 0.02	0.856 ± 0.03	0.791 ± 0.03
thr = 5	0.880 ± 0.02	0.880 ± 0.02	0.676 ± 0.04	0.904 ± 0.02	0.862 ± 0.04	0.804 ± 0.03

**Table 4:** The Specificity score on different RNN cell unit.

	Unidirectional			Bidirectional		
	GRU	LSTM	Simple	biGRU	biLSTM	biSimple
thr = 1	0.898 ± 0.02	0.882 ± 0.02	0.633 ± 0.05	0.913 ± 0.02	0.904 ± 0.02	0.816 ± 0.05
thr = 3	0.909 ± 0.04	0.862 ± 0.03	0.689 ± 0.07	0.936 ± 0.02	0.913 ± 0.02	0.833 ± 0.04
thr = 5	0.880 ± 0.02	0.860 ± 0.02	0.718 ± 0.03	0.918 ± 0.02	0.904 ± 0.01	0.840 ± 0.01

#### Effectiveness of the bidirectional RNN architecture

To verify whether a bidirectional RNN setup would be more suitable for modeling the current task, three sets of bidirectional RNNs using the GRU cell, LSTM cell, and simple cell respectively were put to test. The number of cells used in the bidirectional model setup were 128 in each direction to match the number of total cells used in the unidirectional model setup. The AUROC score is shown in Table 2. Overall, performance improvements can be seen for all model configurations, especially for those using the simple cell. This is a very common case as most of the existing literature dealing with RNN could often obtain better results using the bidirectional architecture, and it has gradually become a standard nowadays. The best performance could be achieved using the bidirectional RNN with the GRU cell among all the experiments. Thus, a bidirectional GRU architecture was adopted in building the AD Assessment Engine.

### Evaluation of the feature sequence generator

To measure the transcription quality of the Feature Sequence Generator, three evaluation metrics were used to evaluate the Feature Sequence automatically generated from Mandarin_Lu dataset, which were Edit Distance (ED), Token Error Rate (TER), and Length Difference (LD). ED, which may also be referred to as Levenshtein distance^45^, measures the number of insertion (of a symbol that is not in the reference string), deletion (of a symbol that is in the reference string), and substitution (of a symbol that is in the reference string with another symbol) between two strings, or two Feature Sequence in our case. TER is a metric modified from Word Error Rate (WER)^46^, where the only difference between the two is that the former works at the token level and the latter works at the word level. TER (WER) can be derived from ED by simply normalize the ED by the length of the reference string. LD is defined as the length difference between two strings. The results are shown in Table 5. Judging from the ED and TER, the performance is far from the state of the art speech recognition models. However, the goal of the Feature Sequence Generator is not a low word error rate model which is required for an ASR task, but a model good enough to represent the key characteristics for identifying speech of an AD patient. It can be seen from LD that the difference is quite small, showing that the Feature Sequence Generator is capable in capturing almost every verbal activity produced by the speaker.

**Table 5:** Evaluation of the Feature Sequence Generator.

	Edit Distance		Token Error Rat		Length Difference	
	CH	AD	CH	AD	CH	AD
Fruit	24.0 ± 7.7	39.6 ± 13.3	0.524 ± 0.129	0.787 ± 0.081	0.7 ± 4.6	-12.1 ± 9.5
Location	33.3 ± 10.6	51.4 ± 19.7	0.426 ± 0.120	0.796 ± 0.099	-1.3 ± 6.7	-13.5 ± 9.7
Picture	73.8 ± 27.7	77.7 ± 30.8	0.448 ± 0.091	0.830 ± 0.056	-12.6 ± 10.7	-28.3 ± 13.8

### NTUH dataset

To evaluate the results of the NTUH dataset, the best Feature Sequence Generator, and the AD Assessment Engine trained using the Feature Sequence generated manually with silence threshold of 3 seconds was applied. To also inspect how tolerable the AD Assessment Engine was without the Feature Sequence being 100% correct and to experiment on whether fine-tuning the AD Assessment Engine using the automatically generated Feature Sequence could improve its performance, automatically generated Feature Sequence was also tested with Assessment Engine. The AUROC score is shown in Table 6. Because of the addition of a new neuropsychological test type, which was much different compared with that conducted in the NTU dataset, and the increased difficulty of the dataset itself, a slight performance drop can be seen compared with NTU dataset. Moreover, a very small performance drop can be also seen when testing the automatically generated Feature Sequence with the model trained on the manually generated Feature Sequence, which shows the AD Assessment Engine is pretty tolerable as it was not highly affected by the wrongly transcribed Feature Sequence. Experiment also show that, after fine-tuning, the performance could even higher than original Assessment engine training and testing on manually generated Feature Sequence.

**Table 6:** Performance on the NTUH dataset.

	Manual	Automatic Transcription	
	N/A	Without Fine-Tuning	After Fine-Tuning
thr = 3, biGRU (AUROC)	0.808 ± 0.05	0.803 ± 0.03	0.838 ± 0.03
thr = 3, biGRU (Sensitivity)	0.736 ± 0.07	0.711 ± 0.05	0.756 ± 0.07
thr = 3, biGRU (Specificity)	0.750 ± 0.05	0.822 ± 0.03	0.764 ± 0.06

## Discussion

In contrast of previous works, the proposed method shows huge potential in real-world application by saving valuable human and time resources. From developer’s standpoint, the system required the least developing and tuning efforts. Furthermore, it is plausible to self-evolve through time by accumulating more and more data. From the practitioner’s standpoint, a system based on neuropsychological tests possess higher credibility, which can certainly be used as a screening tool. As the clinical diagnosis often takes much time, this helps reduce the burden of all the medical staffs and save the valuable resources to those truly in need. From the public health standpoint, the fully-automated and man-free design increase its availability to the general public, which can be easily deployed to every household. Not only can it provide care to more people, but also keep track of one’s mental condition regularly.

While this study has demonstrated promising preliminary results on the effectiveness of our novel design, four main research directions can be focused on for the future studies. First, more data, especially those from the other types of dementia with different pathologic stages should be collected to complete our system so it is capable of assessing all types and stages of dementia. As different types of dementia have different symptoms, new features or new neuropsychological tests may need to be added in order to characterize the different properties. A different data handling and model training strategy may also be required. Second, the quality of the Feature Sequence Generator is still suboptimal. Although it would not completely sabotage the AD Assessment Engine, there is no harm but benefits if its performance could be improved. One can increase the number of training iterations of the Feature Sequence Generator or collect more training data, especially those with similar wordings and accent in Taiwan. Third, one can consider training a separate AD Assessment Engine for each neuropsychological test. Because the form of each neuropsychological test can differ a lot from one another, the token distribution may get too noisy as the training set grows larger, which may make the training of the AD Assessment Engine difficult. Finally, an end-to-end training procedure could be applied to train the Feature Sequence Generator and the AD Assessment Engine jointly as both are ANN-based models. This may further simplify the setup of our system and make it more compact. Additionally, the performance can also be boosted.
